# Development and Validation of a Large Language Model–Based System for Medical History-Taking Training: Prospective Multicase Study on Evaluation Stability, Human-AI Consistency, and Transparency

**DOI:** 10.2196/73419

**Published:** 2025-08-29

**Authors:** Yang Liu, Chujun Shi, Liping Wu, Xiule Lin, Xiaoqin Chen, Yiying Zhu, Haizhu Tan, Weishan Zhang

**Affiliations:** 1 Medical Simulation Center, Shantou University Medical College, No. 22 Xinling Road, Shantou, 515041, China, 86 754-88900459; 2 Department of Medical Physics and Informatics, Shantou University Medical College, Shantou, China

**Keywords:** large language models, medical history-taking, structured evaluation, evaluation stability, human-AI consistency, evaluation transparency, virtual standardized patient, DeepSeek, Qwen, cross-model generalizability

## Abstract

**Background:**

History-taking is crucial in medical training. However, current methods often lack consistent feedback and standardized evaluation and have limited access to standardized patient (SP) resources. Artificial intelligence (AI)–powered simulated patients offer a promising solution; however, challenges such as human-AI consistency, evaluation stability, and transparency remain underexplored in multicase clinical scenarios.

**Objective:**

This study aimed to develop and validate the AI-Powered Medical History-Taking Training and Evaluation System (AMTES), based on DeepSeek-V2.5 (DeepSeek), to assess its stability, human-AI consistency, and transparency in clinical scenarios with varying symptoms and difficulty levels.

**Methods:**

We developed AMTES, a system using multiple strategies to ensure dialog quality and automated assessment. A prospective study with 31 medical students evaluated AMTES’s performance across 3 cases of varying complexity: a simple case (cough), a moderate case (frequent urination), and a complex case (abdominal pain). To validate our design, we conducted systematic baseline comparisons to measure the incremental improvements from each level of our design approach and tested the framework’s generalizability by implementing it with an alternative large language model (LLM) Qwen-Max (Qwen AI; version 20250409), under a zero-modification condition.

**Results:**

A total of 31 students practiced with our AMTES. During the training, students generated 8606 questions across 93 history-taking sessions. AMTES achieved high dialog accuracy: 98.6% (SD 1.5%) for cough, 99.0% (SD 1.1%) for frequent urination, and 97.9% (SD 2.2%) for abdominal pain, with contextual appropriateness exceeding 99%. The system’s automated assessments demonstrated exceptional stability and high human-AI consistency, supported by transparent, evidence-based rationales. Specifically, the coefficients of variation (CV) were low across total scores (0.87%‐1.12%) and item-level scoring (0.55%‐0.73%). Total score consistency was robust, with the intraclass correlation coefficients (ICCs) exceeding 0.923 across all scenarios, showing strong agreement. The item-level consistency was remarkably high, consistently above 95%, even for complex cases like abdominal pain (95.75% consistency). In systematic baseline comparisons, the fully-processed system improved ICCs from 0.414/0.500 to 0.923/0.972 (moderate and complex cases), with all CVs ≤1.2% across the 3 cases. A zero-modification implementation of our evaluation framework with an alternative LLM (Qwen-Max) achieved near-identical performance, with the item-level consistency rates over 94.5% and ICCs exceeding 0.89. Overall, 87% of students found AMTES helpful, and 83% expressed a desire to use it again in the future.

**Conclusions:**

Our data showed that AMTES demonstrates significant educational value through its LLM-based virtual SPs, which successfully provided authentic clinical dialogs with high response accuracy and delivered consistent, transparent educational feedback. Combined with strong user approval, these findings highlight AMTES’s potential as a valuable, adaptable, and generalizable tool for medical history-taking training across various educational contexts.

## Introduction

History-taking is fundamental to clinical practice and one of the clinicians’ most frequently performed tasks [1]. Although technological advances in assessing patients have proliferated, history-taking remains the most crucial, cost-effective technique [2]. Therefore, enhanced training in medical history-taking is crucial for both improving disease diagnosis accuracy [3-5] and fostering the development of competent physicians [6].

Standardized patients (SPs) effectively teach and evaluate history-taking skills by ensuring structured learning experiences. The training process for SPs is rigorous, time-consuming, and resource-intensive [7,8]. Consequently, the availability of qualified SPs is limited [7]. During SP-based teaching and evaluation, subjective factors are an unavoidable influence [9]. Existing literature suggests SP feedback is highly variable in terms of its content and quality [10,11]. These factors pose a significant challenge to implementing effective one-on-one history-taking training.

The rapid evolution of artificial intelligence (AI) technology, especially with the emergence of large language models (LLMs), has demonstrated significant potential in medical education [12-16]. LLMs can act as virtual standardized patients (VSPs) [12,17-19] and create a human-like conversational experience [12,20]. In addition, the web-based system is accessible at any time, allows repeated practice, and significantly reduces teaching costs. However, feedback is the cornerstone of medical education and is crucial for the continuous learning of trainees [21]. Therefore, a system that only provides interactive practice without structured feedback may fail to meet the instructional needs.

Encouragingly, LLMs can also provide instant feedback. A recent single-case study has shown that an LLM-powered VSP can not only provide accurate interactions but also implement structured evaluations with high human-AI consistency for most assessment items, while identifying a subset of items where further alignment could significantly enhance performance [22]. The inherent scalability and personalization capabilities of LLMs may address the inefficiencies and inconsistencies associated with traditional feedback, holding the promise of democratizing high-quality learning experiences. These findings offer preliminary evidence for applying LLMs to evaluate medical history-taking training.

Despite these promising developments, implementing AI as VSPs presents several challenges and technical limitations, particularly in providing real-time educational feedback [23]. First, while some preliminary studies have explored LLMs in medical history-taking evaluation, there remains a gap in research investigating multiple cases of varying difficulty levels across different clinical scenarios. Specifically, given the diversity of disease types in clinical practice and the variations in content and evaluation standards for history-taking across different diseases, further exploration is required to assess their applicability and effectiveness in a broader range of clinical settings.

Second, LLMs may generate “hallucinations,” producing information that appears reasonable but is incorrect [24], leading to different responses to the same query. Even the most advanced models may struggle to handle complex or highly specialized inputs, affecting the accuracy of their outputs. Furthermore, their decision-making process lacks transparency, resulting in users unable to understand the basis on which they draw conclusions, presenting a “black box” problem [25,26]. This black box characteristic raises doubts about their evaluation results. Research shows that the transparency and credibility of evaluation feedback significantly influence learner acceptance, particularly for AI-generated feedback, which requires clear evaluation criteria and frameworks [23].

Moreover, these risks highlight the importance of adding an extra layer of validation, especially for complex teaching tasks that directly affect patient diagnosis and treatment. It is evident that current LLMs cannot yet be solely relied upon in the fields of education and research. Therefore, to identify and prevent these safety risks, developing a systematic assessment system with broad adaptability is particularly important [27]. Consequently, when developing LLM-powered evaluation systems, special attention must be paid to making the evaluation process transparent and standardized. Finally, the stability of LLM-generated evaluations remains to be characterized, which is a critical factor for long-term application in teaching.

To address these gaps, we developed the AI-Powered Medical History-Taking Training and Evaluation System (AMTES). This study describes its development and rigorously evaluates its dialog quality, evaluation stability, human-AI consistency, and transparency across 3 clinical cases of varying complexity. We also validated our design through systematic baseline and cross-model comparisons.

## Methods

### System Design and Implementation of AMTES

#### Overview of AMTES

AMTES is a web-based system developed using the ASP.NET framework (Microsoft) with a Browser/Server architecture. The system seamlessly integrates the DeepSeek-V2.5 (DeepSeek) application programming interface (API), a sophisticated Chinese LLM. This model was selected after demonstrating superior performance for our specific medical dialog tasks in preliminary tests against other domestic models available during the study’s implementation phase, thereby best fulfilling the project’s technical and accessibility requirements. DeepSeek-V2.5 features an advanced architecture with 236 billion parameters and supports an extensive context window of up to 128,000 tokens, enabling robust natural language understanding and complex reasoning capabilities across extended conversations. The AMTES software has been granted the Computer Software Copyright Registration Certificate by the National Copyright Administration of China (Registration No. 14651073).

#### System Modules

**Figure 1.**
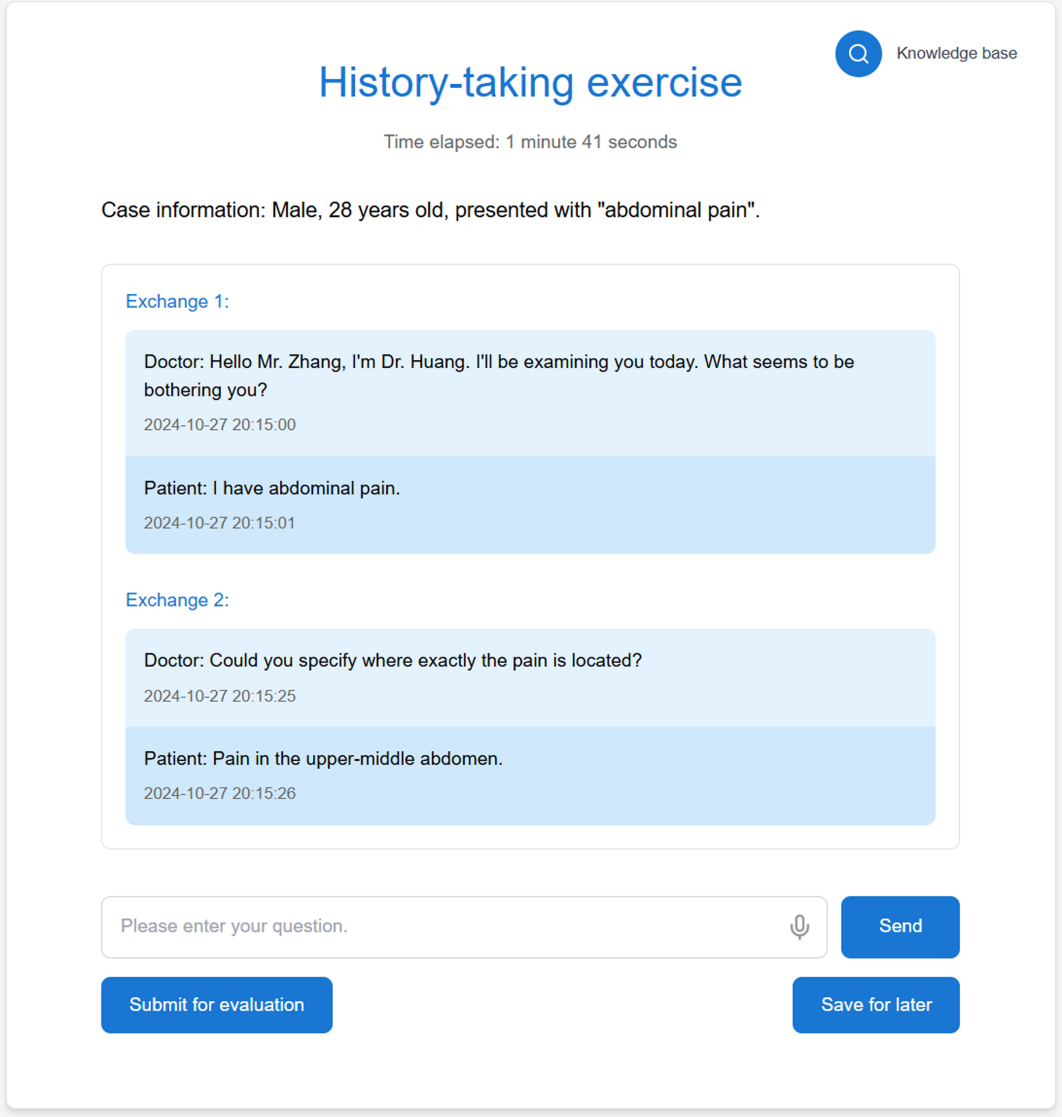
History-taking interface showing the chat environment where students interact with the virtual standardized patient.

AMTES consists of two core modules: (1) the conversational Dialog Module (Figure 1), which enables multiround history-taking conversations with a VSP while recording all interactions for evaluation; (2) the Automated Evaluation Feedback Module, which analyzes dialog records using LLM technology to generate structured feedback. These 2 modules constitute the core of the system: simulation (conversational dialog) and assessment (automated evaluation).

#### System Workflow

**Figure 2.**
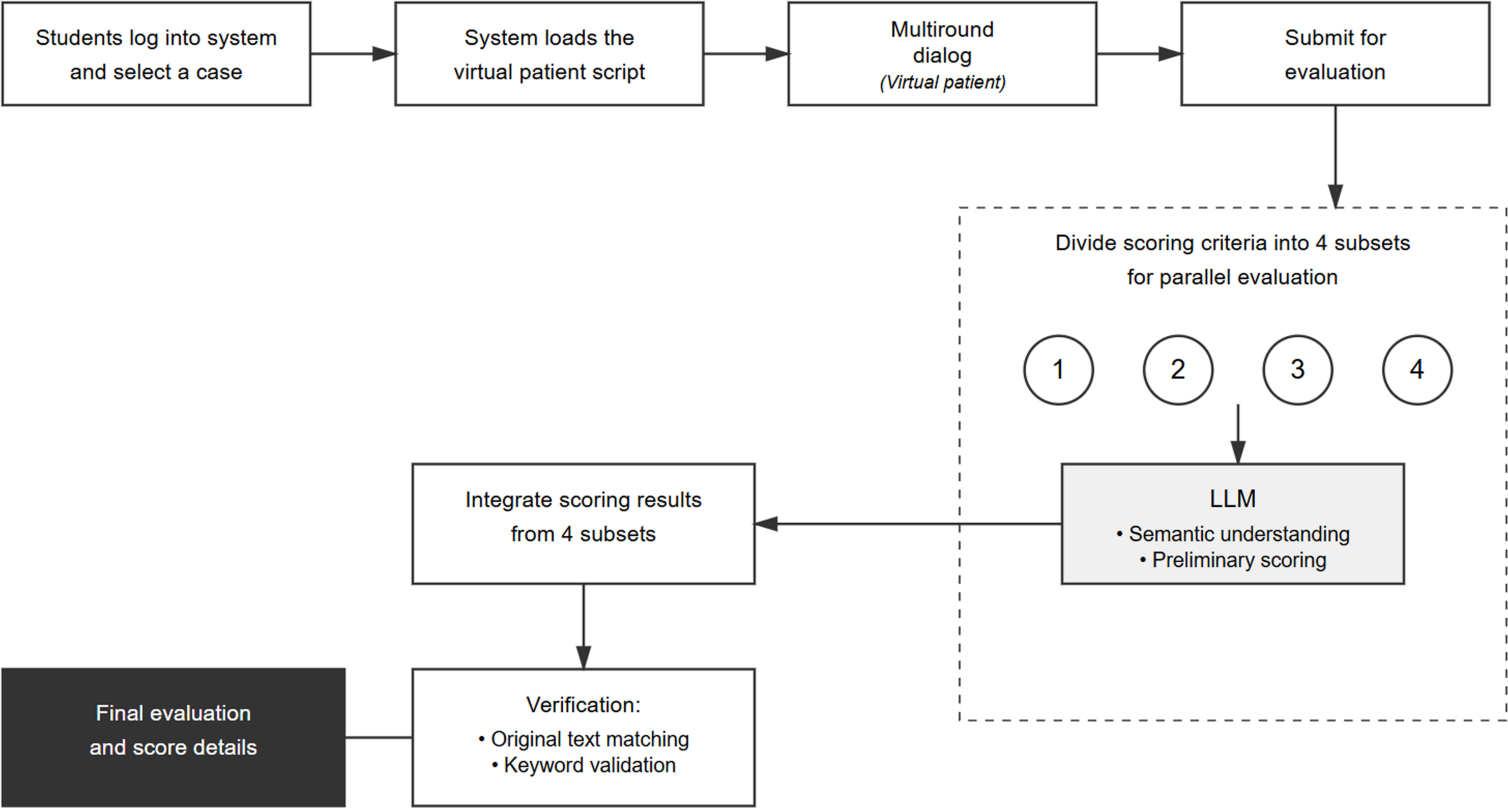
System workflow diagram illustrating the complete medical history-taking training and evaluation process in the Artificial Intelligence–Powered Medical History-Taking Training and Evaluation System. LLM: large language model

The complete system workflow, from student login through final evaluation delivery, is illustrated in Figure 2. A granular, step-by-step description of each stage is provided in Multimedia Appendix 1.

#### System Design Strategies

This subsection elaborates on the underlying principles and innovations guiding AMTES’s development.

#### Framework Objectives and Implementation Strategies

To ensure AMTES meets clinical education requirements, we implemented a comprehensive design framework with specific strategies aimed at achieving system reliability, human-AI consistency, and evaluation transparency (Table 1). System design framework and implementation strategies present this multifaceted framework in a structured format designed to clearly delineate each objective, the strategies used, and their specific implementation methods. These strategies were engineered specifically to address the key LLM challenges of transparency and hallucination identified in the introduction, ensuring the system’s reliability and trustworthiness.

**Table 1.** System design framework and implementation strategies.

Core objective and strategy	Key methods	Specifications and examples
A. Reliability assurance (ensuring accurate and stable system outputs)		
Minimizing randomness	Lower the temperature parameter of the LLM’s^a^ API^b^	Dialog: set temperature as 0.05; assessment: set temperature as 0.0
Implementing multilevel verification^c^	1. Original-text matching: verifies whether the LLM-cited dialog exists verbatim in the original dialog 2. Keyword validation: confirms the presence of predefined mandatory keywords within the cited evidence	The specific strategy is in Multimedia Appendix 2
Parallelizing evaluations^d^	Split scoring items into 4 subsets for parallel LLM queries	Benefits: enables evidence-based scoring by circumventing token limits; reduces evaluation time through parallel processing
B. Human-AI Consistency (aligning AI evaluations with expert judgment)		
Decomposition for standardization	Complex scoring items decomposed into smaller, unambiguous sub-items to improve LLM execution accuracy	For example, original: “persistent moderate-to-severe burning abdominal pain”→ 3 sub-items: Pain is burning in nature Pain is moderate to severe Pain is persistent
Disambiguation via guidelines	Detailed evaluation guidelines for contextual differentiation and terminology clarification	For example, context: “initial dull pain” versus “current burning pain”; terminology: “petechiae”=small red hemorrhagic spots
Few-shot examples	Uses in-prompt examples to demonstrate correct scoring	The complete prompt is available in Multimedia Appendix 3
C. Transparency enhancement (making evaluation processes traceable and verifiable)		
Evidence-based scoring^e^	Prompt engineering compels LLM to cite specific dialog text and explain the rationale for transparent decision-making	The complete prompt is available in Multimedia Appendix 3, “Final Output Format” section
Structured feedback	Organizes feedback hierarchically, from overall metrics down to item-level specifics	Hierarchy: overall performance → category analysis → item details → dialog links

a LLM: large language model

b API: application programming interface

c Multilevel verification mechanism (strategy A) represents a key innovation in handling large language model hallucinations through systematic validation protocols. This approach ensures reliability by implementing multiple checkpoints throughout the evaluation process (see Multimedia Appendix 2 for detailed implementation).

d Parallel evaluation architecture (strategy A) aims to improve computational efficiency while enabling comprehensive evidence-based scoring within token constraints.

e Evidence-based scoring framework ensures full traceability of artificial intelligence decision-making processes through mandatory citation requirements. Every evaluation decision must be supported by explicit dialog evidence.

### Strategy Development and Refinement

The design strategies described above were not developed in isolation but emerged through an iterative development process. Over a 2-month period, a multidisciplinary team comprising 4 clinical instructors, 6 medical students, and 2 engineers extensively tested the system with 3 clinical cases. Through this collaborative process, the AMTES system itself, along with the dialog scripts, prompts, scoring standards, and evaluation guidelines, underwent continuous refinement based on practical insights and user feedback. This iterative approach resulted in a comprehensive set of evaluation rules and a well-structured bank of few-shot examples, establishing a solid foundation for the formal validation studies.

### Construction of the Clinical Case Bank

Three representative cases were prepared by 5 senior clinical experts, aligned with the national medical licensing examination syllabus. They were chosen as the clinical conditions for the simulation due to their relevance to the material being taught at the time. This alignment ensured that the scenario was both clinically pertinent and integrated with the participants’ ongoing coursework in basic sciences and clinical disciplines.

This bank includes 3 cases:

- Case 1: an 18-year-old male presenting with a chief complaint of recurrent cough. (This is a common respiratory system disease, characterized by a short course and typical symptoms. Difficulty: simple.)
- Case 2: a 27-year-old female presenting with a chief complaint of frequent urination. (This case pertains to a common urinary system disease, complicated by patient anxiety and a recurrent history from 6 months previous. Difficulty: moderate.)
- Case 3: a 28-year-old male presenting with a chief complaint of recurrent abdominal pain involving a gastrointestinal system disease. (This case involves a chronic digestive system disease, notable for its recurrent nature and the recent development of complications. Difficulty: complex.)

Each case includes detailed scripts covering the background introduction, patient profile, comprehensive medical history content, and proactively asked questions. The history content comprehensively covers the chief complaint, present illness, past history, personal history, marital history, reproductive history, family history, and, for female patients, menstrual history.

Scoring items were established and points allocated based on the clinical significance and teaching objectives of each medical history segment, with each scoring item clearly corresponding to a single evaluation criterion. The total score is 70 points, with the current medical history accounting for 45‐50 points. Each scoring item is assigned different scores based on its diagnostic value, ranging from 2 points to 0.5 points. The number of scoring items for the 3 cases was 66 for the cough case, 59 for the frequent urination case, and 67 for the abdominal pain case.

### Study Design and Validation Framework

#### Participants

Between September 2024 and November 2024, 31 third-year medical students (16 females, 15 males) from Shantou University Medical College undergoing diagnostic training were recruited. All participants had received theoretical training on medical history-taking. The inclusion criteria were possession of an electronic device and voluntary participation in the teaching trial. All participants provided informed consent, agreed to the use of their data for research purposes, and were provided with a login account with instructions on using AMTES. No participant was excluded from the final analysis.

#### Validation Approach

To rigorously evaluate AMTES, we conducted validation in 3 sequential phases, using 3 complementary strategies to comprehensively assess the system’s performance, stability, and generalizability. Given the pedagogical imperative to protect students’ learning experiences, all participants interacted with the fully processed system. Baseline and generalizability comparisons were conducted retrospectively using stored dialog records, ensuring educational quality while maintaining methodological rigor.

#### Implementation Phases

##### Phase 1 (Weeks 1‐3)

Thrity-one students completed 3 history-taking sessions (cough → frequent urination → abdominal pain) and received immediate feedback from the fully-processed system. Posttraining questionnaires were administered to collect student feedback on system usability.

##### Phase 2

All 93 dialog records were collected, followed by 9 additional rounds of automated assessment to complete the 10 runs required for the comprehensive performance test. Subsequently, teachers not only manually scored the 93 dialog records based on the rubric but also evaluated the quality of the VSP’s conversational dialog responses. The validation work was conducted by 2 senior teachers who were independent of the case development team in order to ensure the reliability of the evaluations.

##### Phase 3

We rescored the same 93 records to perform 2 main analyses. First, a Systematic Baseline Comparison Test was conducted across 3 versions of system: baseline, core-optimized, and fully processed systems. Second, a Cross-Model Generalizability Test was run using the Qwen-Max (version 20250409) model. Since our participants are native Chinese speakers, all interactions with AMTES were conducted in Chinese. The data and screenshots were then translated into English for presentation.

### Validation Strategies Used

The following 3 strategies were used during the implementation phases described above.

#### Comprehensive Performance Test

The fully processed system was executed 10 times per student record to quantify evaluation stability, human-AI consistency, and transparency.

#### Systematic Baseline Comparison

To quantify the contribution of our distinct optimization layers, we conducted a baseline comparison. A 3-level approach was necessary because several of our core strategies are technically interdependent and could not be tested in isolation (eg, our evidence-based scoring strategy, which requires extensive dialog citation, is only feasible through parallel subevaluation to avoid exceeding the LLM’s token limits; in turn, our final postverification stage actively uses this cited evidence to perform its validation checks). Therefore, we designed the study to compare 3 logically sequenced system versions on identical dialog data, representing distinct stages of optimization.

- Baseline system (minimal prompting): this represents the out-of-the-box performance of the LLM, using only a minimal prompt and sequential processing without any of our custom strategies.
- Core-optimized system (enhanced prompt and parallel processing): this version incorporates our full suite of optimization strategies that are applied during the LLM evaluation process. It includes our entire prompt architecture (eg, structured prompts, few-shot examples, evidence-based scoring; see Multimedia Appendix 3 for the complete integrated prompt) and the parallel subevaluation mechanism, which function as a synergistic whole. This stage is designed to isolate the impact of sophisticated prompting and system architecture, but it explicitly excludes any postprocessing of the LLM’s output.
- Fully processed system (with postprocessing verification): this represents the complete, fully processed AMTES system. It builds directly on core-optimized system by adding the final, critical layer of our multilevel verification mechanisms (ie, original-text matching and keyword validation) to the output generated in the previous stage. Comparing this to the previous system allows us to precisely measure the incremental gain achieved by our postprocessing verification strategies.

#### Cross-Model Generalizability Test

To assess the robustness and adaptability of our evaluation framework, we implemented the complete AMTES system using Qwen-Max, an alternative LLM, without modifying any prompts, evaluation strategies, or system parameters. This testing aimed to demonstrate that our design approach could generalize across different LLM platforms, which is crucial for educational institutions that may need to adapt to various AI technologies.

### Outcome Metrics

To rigorously assess AMTES, we defined the following key outcome metrics and their measurement criteria.

#### Stability

This evaluates the consistency of AMTES evaluations across 10 repeated assessments. We calculated the coefficient of variation (CV) for total scores and the counts of scoring items where AMTES’s evaluation matched human scoring (Human-AMTES Matched Item Counts). CV values ≤10% indicated minimal variation, 10%<CV≤20% indicated moderate variation, and CV >20% indicated significant variation.

#### Human-AI Consistency

This refers to the degree of agreement between evaluations by human experts and those generated by the AI system (AMTES) for the same student performance. We measured this consistency at 2 levels:

- Total score level: we assessed consistency by examining the intraclass correlation coefficient (ICC) and Pearson *r* between the overall scores assigned by human experts and the AI system, using human scoring as the benchmark. ICC values ≥0.75 were considered indicative of good reliability, and values ≥0.90 were regarded as highly consistent. For Pearson *r*, values of 0.50‐0.70 were considered moderate, 0.70‐0.90 indicated strong, and >0.90 indicated very strong.
- Item level: for human-AI consistency in item-level scoring, we quantified it using the following metrics.

$$Mean\;Difference\;Items=|Human\;Scoring\;Items-\bar{AI\;Scoring\;Items}|$$

$$Item-Level\;Consistency=\frac{Total\;Items-Mean\;Difference\;Items}{Total\;Items}\times 100\%$$

#### Transparency

Transparency was defined qualitatively per report: every scoring item had to (1) include a verbatim dialog citation with its rationale and (2) pass both stages of automated verification (original-text matching and keyword checks).

### Statistical Analysis

The study data were analyzed using SPSS 24.0 (SPSS Inc). The Shapiro-Wilk normality test was conducted to examine whether the collected data followed a normal distribution. Values were presented as the mean (SD) for normally distributed data. In terms of dialog quality, the one-way ANOVA was performed to determine differences in the accuracy and appropriateness of AMTES responses across the 3 case scenarios. The CV was calculated to measure the system stability. The consistency at the total score level was assessed using the ICC and Pearson *r*. At the item level, the mean difference items and item-level consistency were calculated to evaluate the average discrepancy. Statistical significance for all tests was set at *P*<.05.

### Ethical Considerations

This study received ethical approval from the Ethics Committee of Shantou University Medical College (approval number: SUMC-2024‐079). All procedures were conducted in accordance with the principles of the Declaration of Helsinki and complied with relevant Chinese laws and institutional ethical standards. Prior to participation, all subjects provided written informed consent. Participants were fully informed of the study's purpose, procedures, and data handling methods and were explicitly told that they could withdraw from the study at any time without penalty. To ensure the privacy and confidentiality of participants, all data were anonymized. Personally identifiable information was removed from the research data, and all files were securely stored in password-protected documents accessible only to the research team. No compensation was provided to the participants for their involvement in this study.

## Results

### Participant Demographics

There were 16 (52%) females and 15 (48%) males in this study. All participants were enrolled in the same stage of their diagnostics curriculum and had the experience of learning theoretical knowledge but lacked practical experience in simulated patient history-taking. The study achieved a 100% completion rate, with no dropouts, and all participants were included in the final analysis.

### Analysis of AMTES’s Conversational Dialog Quality

A descriptive analysis was performed on all conversational dialog records between the 31 students and AMTES across 3 clinical case scenarios to assess the quality of AMTES’s conversational dialog performance. The students completed a total of 93 history-taking sessions, generating 8606 questions (cough scenario: 2383; frequent urination: 2818; abdominal pain: 3405). The system’s response rate was 100%.

The accuracy rates (respond in accordance with the case script) of AMTES’s replies were as follows: 98.6% (SD 1.5%) for cough, 99.0% (SD 1.1%) for frequent urination, and 97.9% (SD 2.2%) for abdominal pain. The proportion of contextually appropriate responses from AMTES was consistently high: 99.74% (SD 0.67%) for cough, 99.09% (SD 1.14%) for frequent urination, and 99.36% (SD 1.03%) for abdominal pain.

Despite occasional participant errors, such as spelling mistakes, AMTES demonstrated the ability to accurately interpret and respond to the majority of these questions. Specifically, in the cough scenario, out of 28 erroneous questions, AMTES correctly interpreted and responded to 26; in frequent urination, out of 40 erroneous questions, 32 were correctly handled; and in abdominal pain, 12 out of 15 erroneous questions were correctly addressed by AMTES (Table 2).

**Table 2.** Quality analysis of Artificial Intelligence (AI)–Powered Medical History-Taking Training and Evaluation System conversational dialog.

Case	Cough (n=31)	Frequent urination (n=31)	Abdominal pain (n=31)	*P* value
Response accuracy (%)^a^, mean (SD)	98.60 (1.5)	99 (1.1)	97.90 (2.2)	.04
Number of questions asked, mean (SD)	76.87 (21.22)	90.9 (21.56)	109.84 (28.83)	<.001
Information appropriateness rate (%)^b^, mean (SD)	99.74 (0.67)	99.09 (1.14)	99.36 (1.03)	.03
Number of incorrect student questions, n	28	40	15	—^c^
Number of questions correctly understood and answered by AMTES^d^^e^, n	26	32	12	—

a Response accuracy (%) denotes the proportion of system responses evaluated as entirely correct relative to the total number of valid AI-generated answers in that scenario.

b Information appropriateness rate (%) refers to the percentage of system responses deemed relevant and contextually appropriate to the questions asked.

c Not available.

d AMTES: Artificial Intelligence–Powered Medical History-Taking Training and Evaluation System.

e The number of questions correctly understood and answered by AMTES shows how many of those erroneous questions were still accurately interpreted and properly answered by AMTES.

### AMTES Provided Transparent and Structured Evaluation Reports

**Figure 3.**
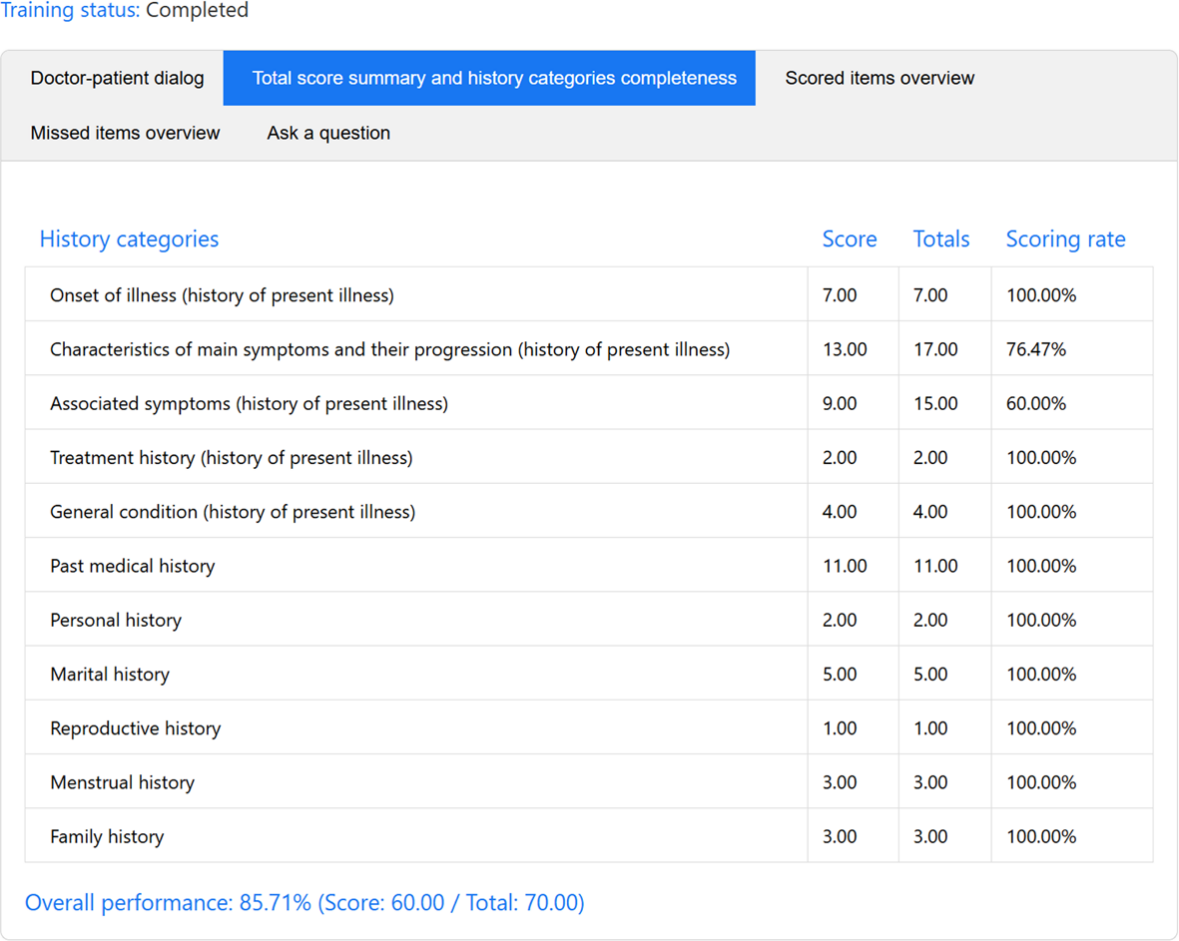
Artificial Intelligence–Powered Medical History-Taking Training and Evaluation System comprehensive feedback report showing the structured evaluation components.

After automatically evaluating each student’s history-taking session, AMTES generated comprehensive feedback reports that included the following components: doctor-patient dialog records, total score per attempt, completeness percentages for each history category, an overview of scored items, and an overview of missed items, thereby comprehensively presenting the evaluation feedback (Figure 3). The history categories included chief complaint, history of present illness, past medical history, personal history, menstrual history, reproductive history, family history, and other relevant sections.

**Figure 4.**
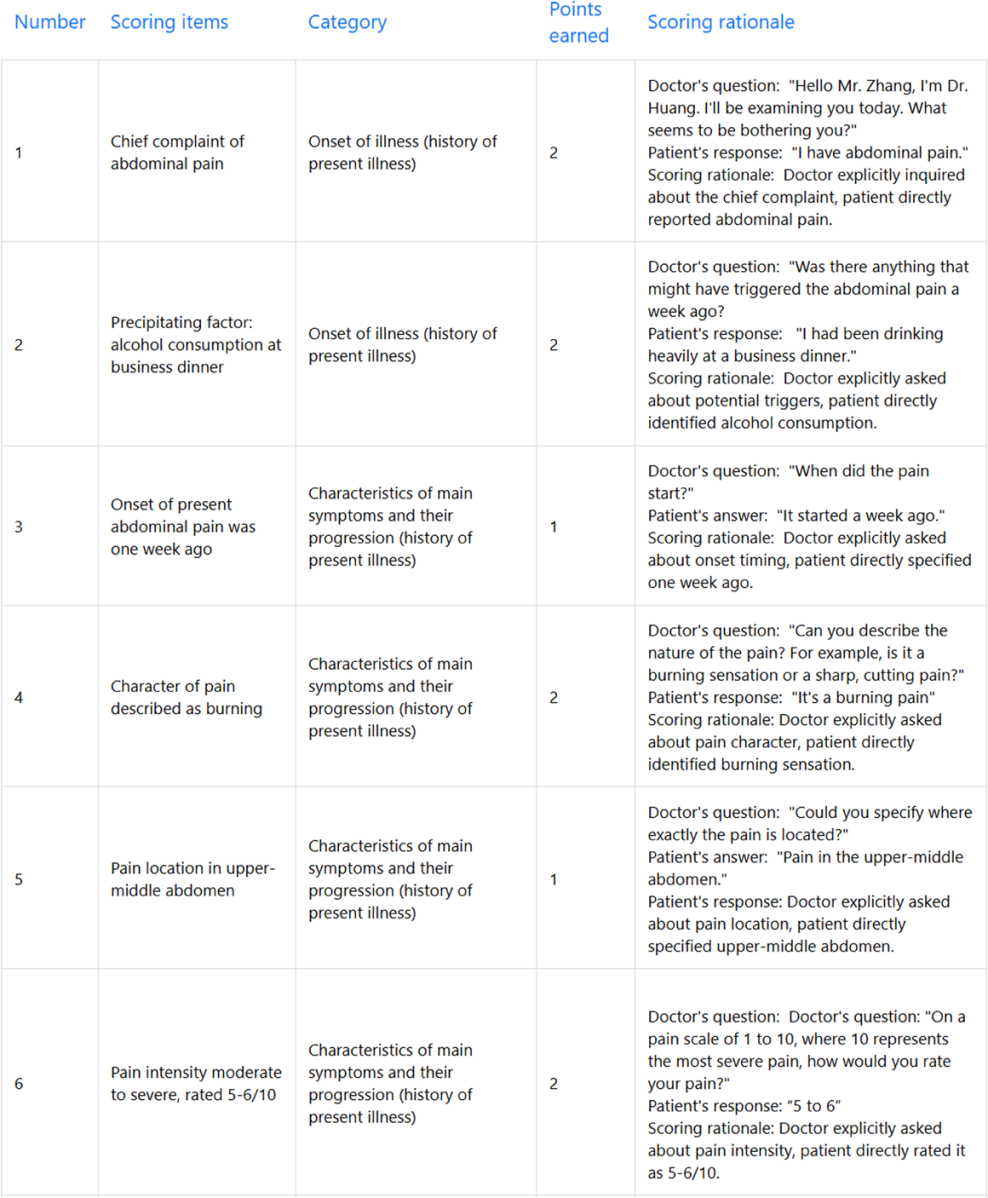
Scoring items and rationale display showing how the system justifies scores.

Importantly, across all 93 history-taking records, every feedback report met the predefined transparency criteria. The scoring items displayed specific scoring criteria with clear rationales, containing verbatim dialog citations along with explicit explanations of how the cited text satisfied the corresponding scoring standard (Figure 4). System logs confirmed that all displayed items passed both the original-text matching and keyword checks; items that lacked direct evidence or failed validation were automatically removed and were never shown to students.

Furthermore, AMTES implemented comprehensive logging of the evaluation process, capturing inputs, outputs, scoring results, and error data for each interaction. This logging facilitated subsequent verification, analysis, and system refinement. Therefore, the AMTES assessment process remained fully traceable and interpretable, providing learners with clear and reliable justifications for each awarded point.

### AMTES Demonstrates High Stability and Repeatability in Evaluations

The stability and reliability of the system were confirmed by consistently low CVs at multiple levels of analysis. At the total score level, the average CVs were 0.87%, 1.12% and 1.07% for cough, frequent urination, and abdominal pain cases, respectively. At the item level, the CVs were exceptionally low, with averages of 0.55% (cough), 0.73% (frequent urination), and 0.67% (abdominal pain) using human evaluations as the benchmark. At the specific history category level, the “chief complaint” category notably achieved a CV of 0 in both the cough and abdominal pain cases, indicating perfect consistency. Even the categories with the highest variability, such as “present history,” maintained very low CVs (eg, 0.65% and 0.95%). These consistently low CV values across all levels of analysis robustly demonstrate that AMTES provides highly stable and reliable structured evaluations. All detailed CV data, including ranges and category-specific breakdowns, are presented in Multimedia Appendix 4.

### Human-AI Consistency in Structured Evaluation

#### Human-AI Consistency at Total Score Level

**Figure 5.**
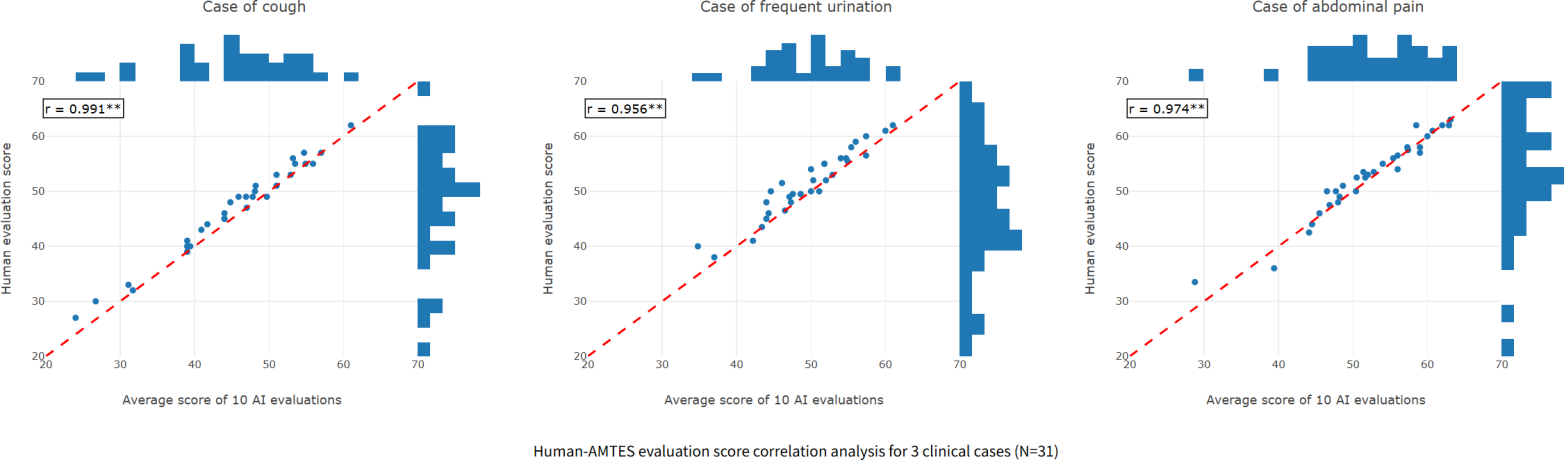
Human-AMTES evaluation score correlation analysis showing strong positive correlations across all 3 cases. AI: artificial intelligence. AMTES: Artificial Intelligence–Powered Medical History-Taking Training and Evaluation System. **P<.01.

Excellent consistency was observed between the total scores assigned by AMTES and human evaluators. The ICC exceeded 0.923 across all 3 clinical scenarios, indicating a high level of agreement between the AI and human experts. This strong positive relationship was further supported by high Pearson *r* (Figure 5). A detailed breakdown of the mean scores, SD, and specific ICC values with 95% CIs for each case is available in Multimedia Appendix 4.

#### Human-AI Consistency at Item Level

The primary metric for evaluating human-AI consistency is the item-level consistency, which directly reflects the proportion of scoring items where AMTES and human evaluators agree. This metric provides a more accurate assessment of evaluation quality than total score comparisons, as it avoids the confounding effect where errors in opposite directions might cancel out. Across all 3 cases, not only was the mean difference items less than 3, but the item-level consistency was also remarkably high, exceeding 95% in all scenarios. Even in the most complex abdominal pain case, the system maintained an item-level consistency rate of 95.75%, demonstrating its robustness in nuanced evaluations (Table 3). Overall, AMTES demonstrated high consistency with human evaluations across multiple case scenarios.

**Table 3.** Discrepancy and consistency analysis of human-AMTES^a^ matched item counts by case groups.

Case	Total items, n	Mean difference items, mean (SD)	Item-level consistency, %
Cough (n=31)	66	1.89 (1.49)	97.13
Frequent urination (n=31)	59	2.06 (1.36)	96.50
Abdominal pain (n=31)	67	2.85 (1.56)	95.75

a AMTES: Artificial Intelligence–Powered Medical History-Taking Training and Evaluation System.

### Student Questionnaire

All 31 distributed questionnaires were returned (response rate: 100%). The results are displayed in Table 4. When considering whether the AMTES is helpful, a large proportion of students agreed (n=14, 45%) or strongly agreed (12, 39%). A significant portion (n=14, 45%) strongly agreed, and 11 (35%) agreed with the notion that the feedback and evaluation are very valuable. Furthermore, 11 (35%) students agreed and 15 (48%) strongly agreed that they would like to use the AMTES in the future. When asked whether they would like to recommend this AMTES to others, 11 (35%) agreed and 17 (55%) strongly agreed, while only 1 (3%) student disagreed and none strongly disagreed.

**Table 4.** Results of student questionnaire feedback.

Item	Students responding (n=31), n (%)				
	Strongly agree	Agree	Neutral	Disagree	Strongly disagree
AMTES^a^ is helpful as an additional tool	12 (39)	14 (45)	4 (13)	1 (3)	0 (0)
Feedback and evaluation are very valuable	14 (45)	11 (35)	5 (16)	1 (3)	0 (0)
Would like to use the AMTES in the future	15 (48)	11 (35)	4 (13)	1 (3)	0 (0)
Would like to recommend this AMTES to others	17 (55)	11 (35)	2 (6)	1 (3)	0 (0)

a AMTES: Artificial Intelligence–Powered Medical History-Taking Training and Evaluation System.

### Baseline Comparison Analysis

The baseline comparison revealed substantial and consistent improvements across multiple dimensions, as detailed in Table 5.

**Table 5.** Baseline comparison of system performance across implementation levels and cross-model generalizability test results using Qwen-Max.

Metric and case	Baseline system	Core-optimized system	Fully-processed system	Relative change (%) baseline→fully-processed	LLM^a^ backend Qwen-Max	Δ (Qwen - DeepSeek)
CV^b^ (%), mean (range)						
Cough (n=31)	2.09 (0‐8.66)	1.78 (0‐5.71)	0.87 (0‐2.78)	−58.3	1.71 (0‐3.93)	+0.84
Frequent urination (n=31)	1.68 (0‐8.22)	3.02 (0‐14.99)	1.12 (0‐7.64)	−33.3	2.90 (1.10‐6.05)	+1.78
Abdominal pain (n=31)	0.57 (0‐4.12)	0.98 (0‐2.96)	1.07 (0‐3.78)	+87.7	2.19 (0.63‐4.91)	+1.12
Mean difference items, mean (SD**)**						
Cough (n=31)	4.83 (2.28)	4.60 (2.28)	1.89 (1.49)	−60.9	2.24 (1.14)	+0.35
Frequent urination (n=31)	7.92 (2.56)	4.83 (2.86)	2.06 (1.36)	−74.0	2.86 (1.59)	+0.80
Abdominal pain (n=31)	9.44 (3.86)	4.05 (2.17)	2.85 (1.56)	−69.8	3.71 (2.18)	+0.86
Item-level consistency, %						
Cough (n=31)	92.69	93.03	97.13	+4.8	96.60	−0.53
Frequent urination (n=31)	86.58	91.82	96.50	+11.5	95.14	−1.36
Abdominal pain (n=31)	85.92	93.96	95.75	+11.5	94.45	−1.30
ICC^c^ (95% CI)						
Cough (n=31)	0.866 (0.785‐0.942)	0.864 (0.747‐0.931)	0.978 (0.955‐0.989)	+12.9	0.970 (0.938‐0.985)	-0.008
Frequent urination (n=31)	0.414 (0.023‐0.702)	0.663 (0.445‐0.815)	0.923 (0.849‐0.962)	+122.9	0.893 (0.792‐0.947)	+0.030
Abdominal pain (n=31)	0.500 (0.044‐0.775)	0.897 (0.803‐0.948)	0.972 (0.943‐0.986)	+94.4	0.973 (0.945‐0.987)	+0.001
Pearson *r*						
Cough (n=31)	0.953	0.944	0.991	+4.0	0.983	−0.008
Frequent urination (n=31)	0.768	0.785	0.956	+24.5	0.969	+0.013
Abdominal pain (n=31)	0.866	0.948	0.974	+12.5	0.973	−0.001

a LLM: large language model.

b CV: coefficients of variation.

c ICC: intraclass correlation coefficient.

#### Enhanced Evaluation Stability

For the cough case, CV saw a substantial 58.3% reduction, moving from a mean of 2.09% (baseline system) to 0.87% (fully processed system), indicating minimal variation. While frequent urination also showed a 33.3% reduction (1.68% to 1.12%), abdominal pain presented a unique trend, with CV increasing from 0.57% (baseline system) to 1.07% (fully processed system), suggesting increased variability in this most complex scenario despite overall alignment gains.

#### Significant Improvements in Human-AI Consistency

The optimization process yielded remarkable gains in human-AI alignment across multiple metrics. For detailed data, please refer to Table 5.

##### Item-Level Consistency

Our optimization efforts yielded significant gains in item-level human-AI consistency. Across all cases, the mean number of discrepant items saw reductions ranging from 60.9% to 74.0%. This was accompanied by a notable rise in the item-level consistency, which climbed from 85.92%‐92.69% (baseline) to a consistently high 95.75%‐97.13% (fully processed system). These improvements consistently showed incremental gains from the baseline system to core-optimized system and then to the final fully processed system (Table 5).

##### Total Score-Level Consistency

Total score-level consistency demonstrated even more striking improvements, with both the ICC and Pearson *r* showing significant positive changes.

The ICC values experienced exceptional growth, particularly as case difficulty increased, showcasing the optimization’s pronounced effect on alignment in more complex scenarios. For instance, the ICC for frequent urination surged by an impressive 122.9% (from 0.414 to 0.923), transforming from weak to highly consistent. Similarly, abdominal pain saw a 94.4% increase (from 0.500 to 0.972), also achieving high consistency. Even for the cough case, ICC improved by 12.9% (from 0.866 to 0.978), reaching near-perfect consistency. The progressive nature of these gains was clear across all versions of the systems (Table 5).

Correlation strength, as indicated by Pearson *r,* consistently improved across all cases. It moved from a strong correlation for cough (0.953) to nearly perfect (0.991), and from moderate and strong to very strong for frequent urination (0.768 to 0.956) and abdominal pain (0.866 to 0.974). This further confirms a much tighter alignment, with noticeable steps of improvement from each optimization layer (Table 5).

##### Cross-Model Generalizability Validation

Upon replacing DeepSeek-V2.5 in the AMTES system with Qwen-Max, the item-level consistency remained high (94.45%‐96.60%), confirming its excellent reliability. Notably, in the more challenging abdominal pain case, it demonstrated high human-AI consistency, with a Pearson *r* reaching 0.969. Despite a slight increase in the average number of differing items (18.5%‐38.8% increase, or 0.35‐0.86 additional items) with Qwen-Max, the absolute difference remained very small, and the item-level consistency rate only saw a minor decrease of 0.53%‐1.36% (Table 5). Therefore, this cross-model validation provides strong evidence supporting the effectiveness of the AMTES system framework.

## Discussion

### Principal Findings and Methodological Innovations

In this study, we successfully developed the AMTES. Our findings demonstrate that through our comprehensive framework of integrated design strategies, AMTES effectively simulates patient interactions across 3 cases of varying difficulty, providing high-quality dialog and transparent, evidence-based feedback. Critically, its evaluations achieved exceptional stability (mean CV <1.20%) and high human-AI consistency (mean ICC >0.923). This remarkable stability and consistency demonstrate that AMTES holds significant potential as a history-taking training tool in medical education. The use of AMTES as a standardized patient offers a more accessible alternative to traditional human standardized patients, potentially enhancing access to medical training, especially in resource-limited settings. The positive student reception further supports its significant potential as an engaging history-taking training tool.

The key to achieving these robust results lies in our systematic approach, which extends beyond conventional prompt optimization to encompass a multistage strategic framework. Pre-evaluation, we implemented “Decomposition for Standardization” to break down complex tasks and “Disambiguation via Guidelines” to ensure input clarity. During the evaluation, we architected a “Parallelizing Evaluations” mechanism. This architecture segments the scoring task into multiple concurrent sub-queries, which not only circumvents token limit constraints in long-context scenarios but also significantly enhances processing throughput. Post-evaluation, a “Multi-level Verification” mechanism was deployed to cross-reference and validate the preliminary results, ensuring the accuracy and reliability of the final output. It is this organic integration of strategies across the entire workflow that provided the foundation for AMTES’s superior performance.

### Empirical Performance Results and Comparison With Previous Work

High-quality doctor-patient interaction is crucial in history-taking training. AMTES addresses the need for patient simulation through the integration of a LLM DeepSeek-V2.5. Through rule restrictions and multiple validations, AMTES mitigated the “hallucination” issue commonly associated with LLMs in complex dialogs, as well as the occurrence of unreliable answers stemming from their strong reasoning abilities, as noted in previous studies [12]. Our results show that response accuracy and information appropriateness are highest in the simplest cases among 3 different levels of difficulty, at 98.6% (SD 1.5%) and 99.74% (SD 0.67%). These response accuracy rates are on par with those of ChatGPT-powered systems [22,28]. In addition, our findings thus confirm that LLM-powered systems exhibit high accuracy and completeness, with accuracy slightly lower for higher difficulty compared to lower difficulty. This observation is consistent with findings from other studies [28].

Experimental data demonstrate that the fully processed system exhibits exceptional stability in repeated structured evaluations across 3 distinct cases. The CVs of total scores are low-cough: 0.87% (range 0%‐2.78%); frequent urination: 1.12% (range 0%‐7.64%); abdominal pain: 1.07% (range 0%‐3.78%). Moreover, the system shows low CVs in both item-level scoring and across history categories. Furthermore, AMTES demonstrates high consistency with human evaluations in both total score level and item-level assessments, thereby confirming the system’s significant reliability and accuracy. This accuracy surpasses that reported for virtual patient systems in previous studies [29-31] and, in some aspects, exceeds the human-AI consistency of ChatGPT-4.0-driven systems [22].

### Optimization Impact and Cross-Model Generalizability

Our baseline comparison analysis provides empirical evidence for the value of systematic optimization in LLM-powered educational tools. The progressive improvements from baseline system to fully processed system, particularly the dramatic enhancements in human-AI consistency for complex cases (ICC improving from 0.500 to 0.972 for abdominal pain), demonstrate that sophisticated prompt engineering and verification mechanisms can transform unreliable LLM outputs into clinically acceptable evaluations. Most notably, the differential impact across case complexities, with the greatest improvements observed in the most challenging scenarios, suggests that our optimization strategies are particularly valuable for nuanced clinical assessments where raw LLMs struggle most. These findings offer practical guidance for institutions, rather than accepting out-of-the-box LLM performance, investing in comprehensive optimization can yield evaluation tools that approach human-level consistency.

Beyond demonstrating the importance of optimization, our framework also exhibits remarkable cross-model adaptability. Validation experiments with the new large-language model Qwen-Max demonstrated that, without any prompt modifications, our system could still provide transparent, stable, and highly accurate structured evaluations. The success of this experiment challenges the common assumption that LLM-powered systems require extensive customization for each model. This finding indicates that a well-designed evaluation framework can achieve a level of abstraction that transcends specific model architectures, and its cross-model generalizability suggests great potential for medical education applications. Educational institutions often face constraints in technology choices due to institutional policies, regional regulations, or resource limitations. Although our preliminary findings suggest that the evaluation framework may not be entirely dependent on a specific LLM, further validation across diverse platforms is needed to confirm this architectural flexibility. If fully realized, such adaptability could potentially facilitate broader adoption of AI-driven educational tools in varied educational settings. These findings suggest that a one-size-fits-all approach to implementing LLMs in education is suboptimal; instead, investing in a structured, multi-layered optimization and verification framework is critical to unlocking their full potential as reliable assessment tools.

### Educational Value and Student Feedback

Evaluation and feedback are critical in clinical education [32], and particularly structured and procedural assessments, which positively impact teaching and student learning [33]. Therefore, through continuous prompt optimization, our fully processed system not only outputs structured scores but also provides detailed rationales for each item-level score by citing specific dialogs from the text, addressing the inherent opacity of scoring reasons in traditional SP programs and existing virtual patient systems. By providing clear evidence for each scoring decision, AMTES helps students understand not only what they missed but also why specific items are important for comprehensive history-taking. This transparency is crucial for building trust in AI-based educational tools and supporting effective learning.

Students who participated in the study provided positive feedback. Among them, 11 (35%) students agreed and 14 (45%) strongly agreed that the system’s evaluation function is valuable. 11 (35%) students agreed and 15 (48%) strongly agreed to continue using the AMTES system for history-taking practice. Moreover, the majority of students were willing to recommend the system to their peers. The strong performance of AMTES across 3 cases of different difficulties and disease types, along with positive user feedback, highlights its potential adaptability to a broader range of clinical scenarios. This matches studies showing that AI in health care can help develop communication skills, critical thinking, and clinical reasoning abilities through good interactions and clear feedback [34]. In addition, studies confirm that practice in virtual environments helps improve skills and confidence in real-world clinical encounters [35,36], suggesting that tools like AMTES can optimize educational resources while maintaining educational quality.

### AMTES Positioning and Application Prospects

Specifically, AMTES breaks through traditional training resource limitations and accessibility barriers by offering 24/7 learning support, personalized learning experiences, and tailored-structured feedback independent of standardized patient availability. This continuous accessibility and tailored response capability are key strengths of LLMs in medical education [37], potentially supporting student learning efficiency and skill acquisition. AMTES represents a significant advancement in history-taking education, but it is designed to complement rather than replace traditional standardized patient (SP) training. Given the focus of AMTES on evaluating the completeness of history-taking, it is evident that AMTES excels at providing continuous availability for early-stage skill development, allowing students to practice at their own pace and receive consistent, item-level feedback throughout their learning journey. However, the system currently lacks the ability to simulate nonverbal communication (such as facial expressions and body language) and cannot provide emotional understanding and ethical guidance in its feedback - elements which are unique strengths of human SP interactions. While LLMs demonstrate decision-making capabilities, their potential as a replacement for evidence-based professional teaching remains to be fully explored [37]. Therefore, the successful integration of LLMs in medical education feedback, as exemplified by AMTES, is unlikely to lead to the complete replacement of human educators; instead, it may facilitate a redistribution of human effort to areas where it’s most impactful [38].

### Limitations and Future Prospects

We acknowledge that this study has several limitations, which, in turn, provide clear directions for our future research.

First, from a methodological perspective, a key limitation is the retrospective nature of our baseline comparisons. This was a deliberate ethical choice, grounded in the pedagogical imperative to protect the student learning experience. Exposing learners to a potentially unoptimized baseline system risked undermining their motivation and trust, so we prioritized providing all participants with the most reliable and educationally beneficial version of the system. However, we acknowledge this precludes a direct, prospective comparison of learning outcomes between the different system versions. Future research could use a randomized controlled trial design to provide more definitive evidence on the educational impact of each optimization layer.

Second, the current system’s inability to simulate or interpret nonverbal communication (eg, facial expressions and body language) represents a significant shortcoming in achieving the full fidelity of patient-physician interactions. Maintaining a warm and friendly communication style and expressing empathy are particularly crucial for establishing effective doctor-patient relationships. Empathy, as a core competency in doctor-patient interactions [39], has proven highly effective in improving patient satisfaction, treatment outcomes [40], and generating positive health care results [41,42]. To address this gap, future work will focus on integrating cutting-edge multimodal technologies, such as virtual reality (VR), augmented reality (AR), and expressive speech synthesis, to create a more holistic and immersive simulation environment.

Finally, the scope of our validation needs to be broadened. The current evaluation was conducted not only with a limited sample size but was also confined to 2 prominent Chinese LLMs (DeepSeek and Qwen). To comprehensively establish the generalizability of our framework, a crucial future endeavor will be 2-fold: first, to expand our validation dataset with more diverse cases from multiple institutions; and second, to apply our framework to leading international models (eg, the GPT series [OpenAI], Claude [Anthropic]) and evaluate it in different linguistic contexts, such as English.

By systematically addressing these limitations, we are confident that the system has the potential to evolve into a more robust and comprehensive next-generation tool for medical education.

### Conclusion

AMTES, built on a framework of transparent and verifiable evaluation, achieves high stability and human-AI consistency. To our knowledge, this is the first study to systematically evaluate an LLM-powered history-taking evaluation system across multiple disease scenarios while providing empirical validation of design strategies through baseline comparisons and demonstrating cross-model generalizability. By providing students with consistent, evidence-based feedback, AMTES is positioned as a valuable complementary tool in medical education, though further validation in diverse settings would strengthen these conclusions.

## Supplementary material

10.2196/73419 Multimedia Appendix 1 The Artificial Intelligence–Powered Medical History-Taking Training and Evaluation System workflow.

10.2196/73419 Multimedia Appendix 2 Multilevel verification.

10.2196/73419 Multimedia Appendix 3 Comprehensive prompt for medical history-taking scoring.

10.2196/73419 Multimedia Appendix 4 Detailed evaluation metrics.

## Abbreviations

AI: artificial intelligence
AMTES: Artificial Intelligence–Powered Medical History-Taking Training and Evaluation System
CV: coefficient of variation
ICC: intraclass correlation coefficient
LLM: large language model
SP: standardized patient
VSP: virtual standardized patient
