# Supplementary material for: Development and Validation of a Large Language Model–Based System for Medical History-Taking Training: Prospective Multicase Study on Evaluation Stability, Human-AI Consistency, and Transparency
Source: JMIR Med Educ. 2025 Aug 29;11:e73419. doi: 10.2196/73419 (PMC12396829; doi:10.2196/73419)
Supplement: Multimedia Appendix 1 [file mededu-v11-e73419-s001.pdf]

Multimedia Appendix 1: The AMTES workflow

The AMTES workflow consists of nine sequential steps that guide students through the complete history-taking training and evaluation process:

1.Students log into the system and select a case

Students authenticate through the system interface and select a case from the available training scenarios to begin their practice session.

2.System loads the virtual patient script

Upon case selection, the system loads the corresponding virtual patient configuration from the database. This includes:

○Case background information (name, age, gender, chief complaint)

○Structured patient script (CasePrompt) containing:

- ■Role definition for the LLM to portray the specific patient
- ■Complete medical history covering all standard components
- ■Dialogue example templates for response consistency
- ■Behavioral rules governing response scope and length

The system sends an initialization prompt to DeepSeek-V2.5 and awaits confirmation before proceeding.

3.Multi-round dialogue

Students conduct the history-taking through natural language conversation with the VSP. Each question-answer pair constitutes a complete dialogue turn. The system maintains a comprehensive conversation history log, recording all interactions with timestamps and role identifiers for context-aware response generation.

4.Submission for Evaluation

When students determine they have collected sufficient information, they submit their dialogue for automated evaluation.

5.Divide scoring criteria into 4 subsets for parallel evaluation.

To optimize processing efficiency and maintain evaluation quality, the system divides the complete scoring criteria into four subsets for parallel evaluation by the LLM.

6.LLM Semantic Analysis and Preliminary Scoring.

The LLM analyzes each scoring item against the dialogue transcript, identifying whether students obtained the required information and citing specific dialogue evidence for each scoring decision.

7.Integrate scoring results from 4 subsets.

Results from all four parallel evaluation processes are integrated to form a comprehensive preliminary scoring list with supporting rationales.

8.Verification

The system validates the LLM's preliminary scoring results through multi-level

checks to ensure accuracy and reliability before generating the final evaluation.
9.Final evaluation and score details

AMTES generates a comprehensive evaluation report including the complete dialogue transcript, total score, category completion rates, detailed scoring rationales with evidence, and missed items for student review.
