# Supplementary material for: Development and Validation of a Large Language Model–Based System for Medical History-Taking Training: Prospective Multicase Study on Evaluation Stability, Human-AI Consistency, and Transparency
Source: JMIR Med Educ. 2025 Aug 29;11:e73419. doi: 10.2196/73419 (PMC12396829; doi:10.2196/73419)

*Multimedia Appendix 2:*AMTES Systematic Implementation Framework and Strategies

During iterative testing and refinement of AMTES, we identified two critical scoring errors: (1) hallucinations—LLM fabricating nonexistent dialogue, and (2) unwarranted inferences—drawing incorrect conclusions from incomplete evidence. To ensure objective evaluation, AMTES requires verbatim dialogue citation and systematic scoring justification through two complementary validation procedures. These multi-level checks ensure only verifiable, evidence-based scoring items are retained, effectively mitigating these issues while maintaining evaluation objectivity and accuracy.

Original-Text Matching Validation

The LLM outputs preliminary scoring items with item labels, doctor-patient Q&A pairs, and scoring rationales. AMTES then:

1. Text Formatting: Removes extraneous symbols, special characters, and extra spaces from both LLM output and original transcript to prevent mismatches that could undermine evaluation reliability.
2. Exact Matching: Validates each Q&A pair against the original transcript through character-level comparison.
3. Error Correction: Rejects items referencing non-existent dialogue as fabrications.

Example: If LLM cites "Doctor: Have you taken any medication recently? Patient: No" for Item 29, but this exchange doesn't exist verbatim in the transcript, the system logs: "Item Rejected: Item 29, Dialogue Valid: False."

Keyword-Based Validation

For items prone to unwarranted reasoning (e.g., associated symptoms, medical history), AMTES:

a. Keyword Definition: Predefines mandatory keywords using AND/OR operators based on scoring criteria.
b. Evidence Verification: Confirms keyword presence in the cited dialogue evidence.
c. Error Correction: Rejects items lacking required keywords as unsupported inferences.

Example: If LLM scores "no surgery history" based on "Doctor: Have you seen a doctor? Patient: No" with reasoning "surgery requires seeing a doctor," but "surgery" never appears in the dialogue, the system logs: "Item Rejected: Item 40, Keywords Valid: False."

Multi-Level Validation and Correction Flowchart showing the process of verifying LLM scoring decisions.

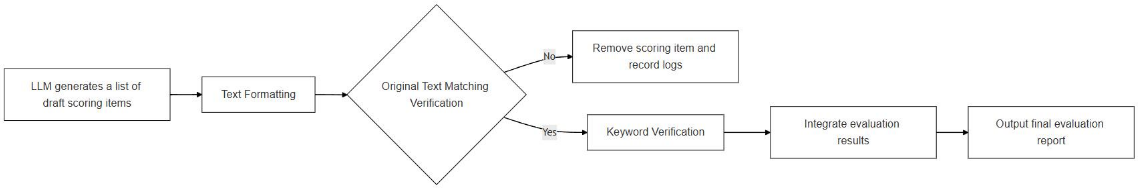

Supplement: Multimedia Appendix 2 [file mededu-v11-e73419-s002.pdf]
