# Supplementary material for: Development and Validation of a Large Language Model–Based System for Medical History-Taking Training: Prospective Multicase Study on Evaluation Stability, Human-AI Consistency, and Transparency
Source: JMIR Med Educ. 2025 Aug 29;11:e73419. doi: 10.2196/73419 (PMC12396829; doi:10.2196/73419)
Supplement: Multimedia Appendix 4 [file mededu-v11-e73419-s004.pdf]

Multimedia Appendix 4: Detailed Evaluation Metrics.

Table 1. The CV and ICC for AMTES evaluation by case groups.

Case Groups	CV of total scores (%), mean(range)	Item-level coefficient (%), mean (range)	ICC, mean (95% CI)
Cough (n=31)	0.87 (0-2.78)	0.55 (0-1.58)	0.978 (0.955-0.989)
Frequent urination (n=31)	1.12 (0-7.64)	0.73 (0-3.78)	0.923 (0.849-0.962)
Abdominal pain (n=31)	1.07 (0-3.78)	0.67 (0-1.58)	0.972 (0.943-0.986)

Table 2. The CV of Human-AMTES matched item counts by history categories in 10 repeated evaluations.

The CV of history category	Cough (n=31)	Frequent urination (n=31)	Abdominal pain (n=31)
The CV of chief complaint (%); mean (range)	0 (0-0)	0.49 (0-15.06)	0 (0-0)
The CV of present history (%); mean (range)	0.65 (0-2.27)	0.33 (0-1.67)	0.95 (0-2.39)
The CV of past history (%) ; mean (range)	0.33 (0-6.27)	0.41 (0-6.87)	0.13 (0-3.90)
The CV of personal history (%); mean (range)	0.25 (0-7.82)	0.53 (0-16.64)	0.50 (0-7.71)
The CV of family history & others (%); mean (range)	0 (0-0)	0.22 (0-4.29)	0.75 (0-4.79)

Table 3. Comparison of AMTES and human total scores across clinical cases.

Case groups	AMTES score (Mean ± SD)	Human score (Mean ± SD)
Cough (n=31)	45.42±8.86	46.81±8.50
Frequent urination	49.62±6.22	51.3(±6.10

(n=31)		
Abdominal pain (n=31)	52.21±7.54	52.73±7.25
